# Synthetic CpG-ODN rapidly enriches immune compartments in neonatal chicks to induce protective immunity against bacterial infections

**DOI:** 10.1038/s41598-018-36588-6

**Published:** 2019-01-23

**Authors:** Thushari Gunawardana, Khawaja Ashfaque Ahmed, Kalhari Goonewardene, Shelly Popowich, Shanika Kurukulasuriya, Ruwani Karunarathna, Ashish Gupta, Betty Lockerbie, Marianna Foldvari, Suresh K. Tikoo, Philip Willson, Susantha Gomis

**Affiliations:** 1 Department of Veterinary Pathology, Western College of Veterinary Medicine, University of Saskatchewan, Saskatoon, SK S7N 5B4 Canada; 2 School of Pharmacy, University of Waterloo, 200 University Avenue West, Waterloo, ON N2L 3G1 Canada; 3 Vaccinology and Immunotherapy, School of Public Health, University of Saskatchewan, Saskatoon, SK S7N 5E3 Canada; 4 Canadian Centre for Health and Safety in Agriculture, University of Saskatchewan, Saskatoon, SK S7N 5E5 Canada

## Abstract

Oligodeoxynucleotides containing CpG motifs (CpG-ODN) induce innate immunity against bacterial infections. Despite recent advances, how CpG-ODN alone protects against bacterial infections remained elusive. Here, we report for the first time, to our knowledge, that CpG-ODN orchestrates anti-microbial protective immunity by inducing a rapid enrichment of various immune compartments in chickens. In this study, eighteen-day-old embryonated eggs were injected with either 50 µg of CpG-ODN or saline (~n = 90 per group). In the first experiment, four days after CpG-ODN treatment, chicks were challenged subcutaneously with a virulent strain of *Escherichia coli* (*E*. *coli*) and mortality was monitored for 8 days. We found significant protection, and reduced clinical scores in CpG-ODN treated chicks. To gain insights into mechanisms of protection induced by CpG-ODN, first we investigated cytokine expression kinetics elicited by CpG-ODN. The spleen and lung were collected from embryos or chicks (n = 3–4 per group) at 10 time points post-CpG-ODN inoculation. Multiplex gene analysis (interleukin (IL)-1, IL-4, IL-6, IL-10, IL-18, interferon (IFN)-γ, IFN-α, and lipopolysaccharide induced tumor necrosis factor (LITAF), revealed a significantly higher expression of pro-inflammatory cytokines following CpG-ODN treatment compared to the saline controls. In our study, LITAF stands out in the cytokine profiles of spleen and lungs, underscoring its role in CpG-ODN-induced protection. The third experiment was designed to examine the effects of CpG-ODN on immune cell populations in spleen, lungs, and thymus. Flow cytometry analysis was conducted at 24, 48 and 72 hrs (thymus only collected at 72 hr) after CpG-ODN administration to examine the changes in CD4^+^ and CD8^+^ T-cell subsets, monocyte/macrophage cell populations and their expression of maturation markers (CD40 and CD86). Flow cytometry data indicated a significant enrichment of macrophages, CD4^+^ and CD8^+^ T-cell subsets in both spleen and lungs of CpG-ODN treated embryos and chicks. Macrophages in spleen and lungs showed an upregulation of CD40 but not CD86, whereas thymocytes revealed significantly high CD4 and CD8 expression. Overall, the present study has demonstrated that CpG-ODN provides protection in neonatal chicks against *E*. *coli* infection not only by eliciting cytokine responses and stimulating immune cells but also through enriching immunological niches in spleen and lungs.

## Introduction

Synthetic oligodeoxynucleotides containing CpG-motifs (CpG-ODNs) are short segments of DNA representing a type of pathogen-associated molecular pattern (PAMP) that stimulate immune cells and trigger immune responses to infections^1–5^. CpG-ODNs act as immunostimulatory agents in many vertebral species including mice^6^, human^7–9^, fish^10^, cattle and sheep^11^, and chickens^12–14^. CpG-ODNs are recognized by toll-like receptors (TLR); TLR-9 and TLR-21 in mammals and avians, respectively. Human TLR-9 and avian TLR-21 are very similar in CpG-ODN motif recognition; and both respond to CpG-ODNs containing GTCGTT motifs, whereas murine but not human TLR-9 recognizes GACGTT motifs^15,16^. Human TLR-9 and avian TLR-21 have similar intracellular localization, signaling cascades, immune activation, and cytokine induction^15,17–19^. Studies reported that CpG-ODNs support innate and adaptive immunity by activating immune cells and inducing cytokine secretion^20^. CpG-ODNs have great promise as vaccine adjuvants and immunotherapeutic agents against infectious diseases and cancer^20–23^.

Our lab has provided the first *in vivo* evidence for an immunoprotective effect of CpG-ODN against bacterial infection in chickens^24^. We demonstrated protection against *E*. *coli* infection in chickens administered CpG-ODN via subcutaneous, intramuscular^24^ or *in ovo* routes^12^. We recently reported that intrapulmonary delivery of CpG-ODN micro-droplets could also protect neonatal chicks against *E*. *coli* septicemia in a dose-dependent manner^25^. The immunoprotective effect of CpG-ODN in chickens was further supported by the studies demonstrating protection against *Salmonella* Enteritidis infection following CpG-ODN administration via intraperitoneal^26^ or *in ovo*^27^ routes. We also demonstrated protective effects of CpG-ODN against *Salmonella* Typhimurium infection^14,28^. The immune protective effect of CpG-ODN can further be improved by formulating it with nanoparticles^28–30^. CpG-ODN was shown to stimulate strong induction of IL-6 and nitric oxide secretion in chicken macrophage cell line (HD11 cells) leading to an increased intracellular killing of *Salmonella* Enteritidis in the activated HD11 cells^31^. CpG-ODN can activate several signaling pathways including protein kinase C (PKC), NF-kappaB and mitogen-activated protein kinases (p38 MAPK and MEK1/2)^32^. Another study reported a significant increase in the heterophil degranulation and oxidative burst following an intraperitoneal administration of CpG-ODN that caused an enhanced resistance to *Salmonella* Enteritidis infection in neonatal chickens^33^. The resistance to intracellular bacterial infection may be the result of a Th1 biased immune response in chickens that was recently reported to be elicited by CpG-ODN^34^. Several recent studies showed enhanced expression of cytokines and chemokines in response to CpG-ODN in chickens^30,35^. Despite these advances, the mechanisms by which CpG-ODN alone provides protection in chickens against bacterial infections are not completely understood. A greater understanding of cellular and molecular mechanisms for CpG-ODN induced antimicrobial immunity will help in harnessing the full therapeutic potential of CpG-ODNs against diseases.

In the present study, we investigated immunity induced by *in ovo* administered CpG-ODN including response to *E*. *coli* challenge, cytokine kinetics by using QuantiGene Plex (multiplex assay for mRNA gene expression), and effects on the immunological niches^36^ in spleen and lungs using flow cytometry.

## Results

### *E*. *coli* challenge

**Figure 1:**
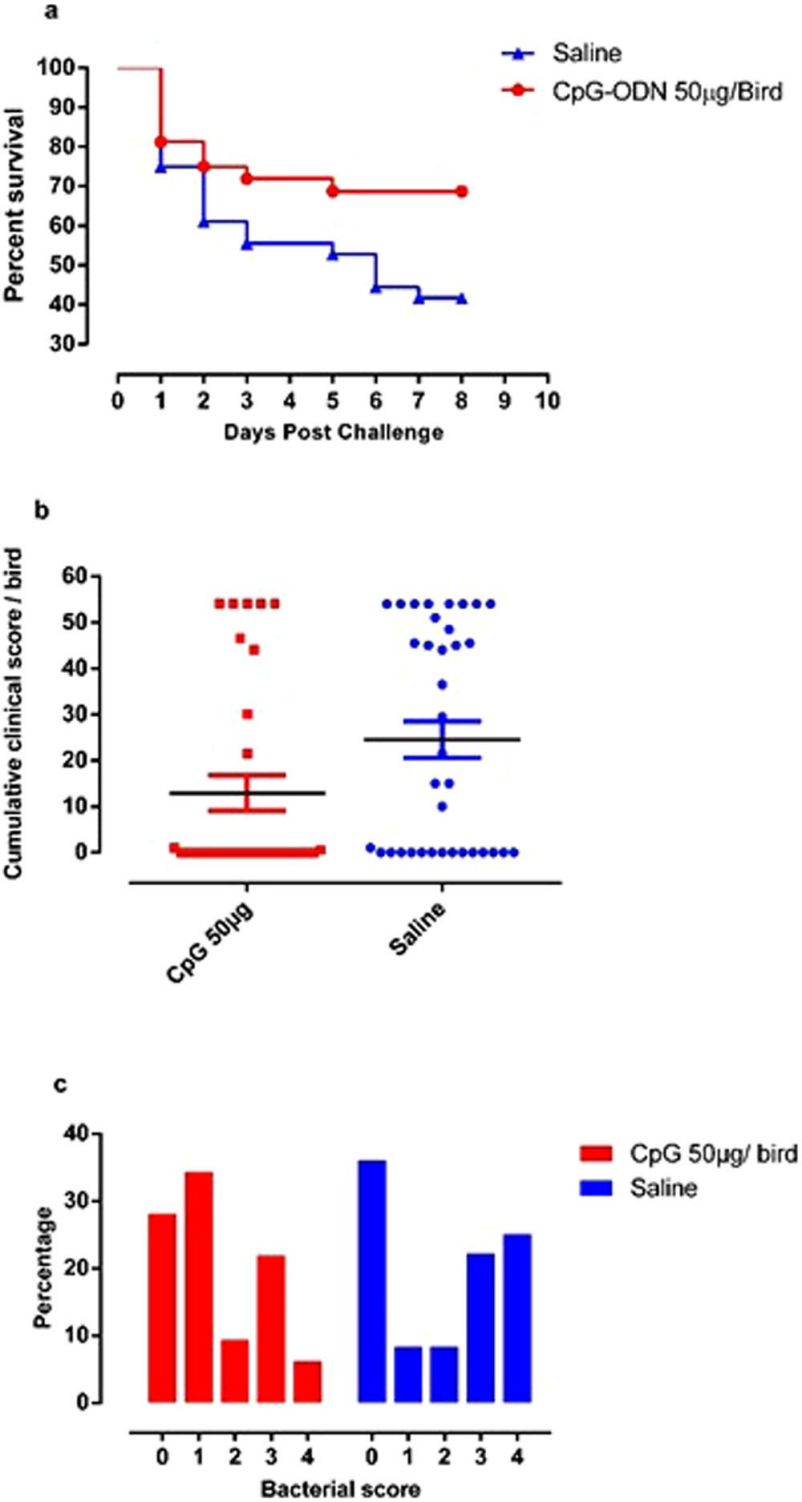
Survival percentages, CCS and bacterial scores. (**a**) Survival of broiler chickens following *E*. *coli* challenge. Groups of broiler chicken embryos at day 18 of incubation were injected with 50 µg of CpG-ODN or saline by the *in ovo* route (n = 32 CpG-ODN, n = 36 saline group). Birds that received CpG-ODN were significantly protected against *E*. *coli* infection compared to the saline control group (P = 0.04). (**b**) Cumulative clinical scores (CCS) of broiler chickens following *E*. *coli* challenge with either 1 × 10^4^ or 1 × 10^5^ cfu of *E*. *coli*. Groups of birds that received 50 µg CpG-ODN CpG-ODN had a significantly lower CCS compared to the saline group, (P = 0.03). Bar = mean. (**c**) Bacterial scores of swabs taken from airsacs of birds. Higher bacterial scores indicate greater bacterial load. Increased bacterial growth was observed more frequently in lesions from birds in the saline group than in the CpG-ODN group. The substantial number of ‘0’ or ‘no growth’ of bacteria in both groups is a common finding in such experiment.

Survival following *E*. *coli* challenge was significantly higher in groups of birds that received 50 µg of CpG-ODN compared to the saline control group (P = 0.04) (Fig. 1a). The cumulative clinical scores (CCS) of chicks following *E*. *coli* challenge with either 1 × 10^4^ or 1 × 10^5^ cfu of *E*. *coli* showed that the chicks that received 50 µg CpG-ODN had a significantly lower CCS compared to the saline group (P = 0.03) (Fig. 1b). Samples collected from the challenged chicks revealed heavy bacterial growth more frequently in samples from chicks in the control group than in the CpG-ODN group (Fig. 1c). Moreover, the reduction in the relative risk of mortality following *E*. *coli* challenge was 46.4% compared to the saline control group. The data of groups that received 1 × 10^4^ and 1 × 10^5^ cfu of *E*. *coli* were combined for clarity of presentation and analysis.

### Gene expression analysis

**Figure 2:**
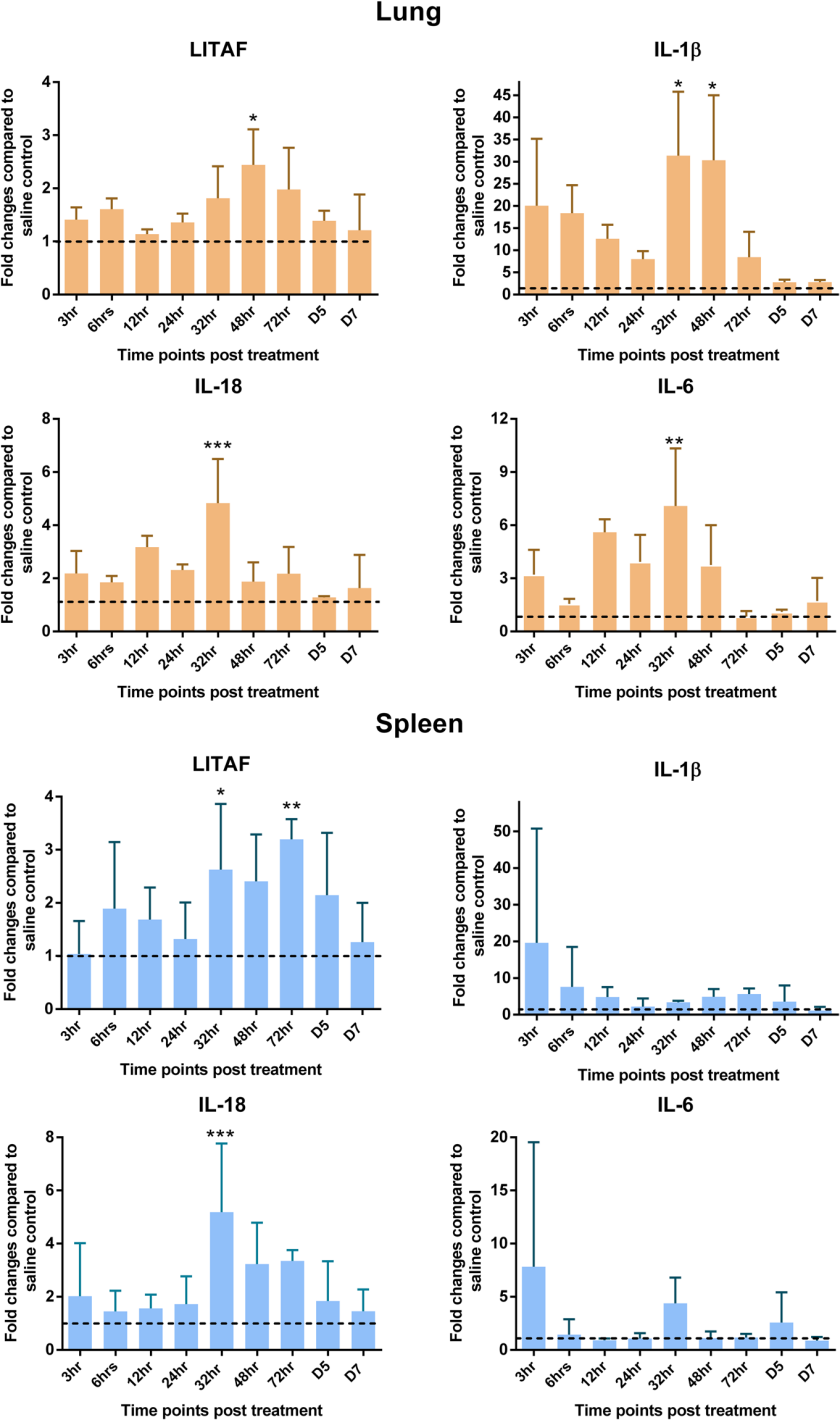
Pro-inflammatory cytokine profiles in lungs and spleen. Mean Fluorescence Intensity (MFI) of each gene was normalized to the average of housekeeping genes (HKGs - Hprt 1 and Tubb 1). Then fold changes were calculated against the saline control at each time point and the means were graphed. (Dotted line shows a fold change of one, which indicates no change compared to the control group and the vertical line and horizontal bar show the standard error of mean-SEM). Dunnett’s test following ANOVA testing was used to test for significant differences of gene expression between CpG-ODN group and the saline control group at each time point. Asterisks indicate groups that were significantly different from control group, *P < 0.05, **P < 0.01 and ***P < 0.001.

**Figure 3:**
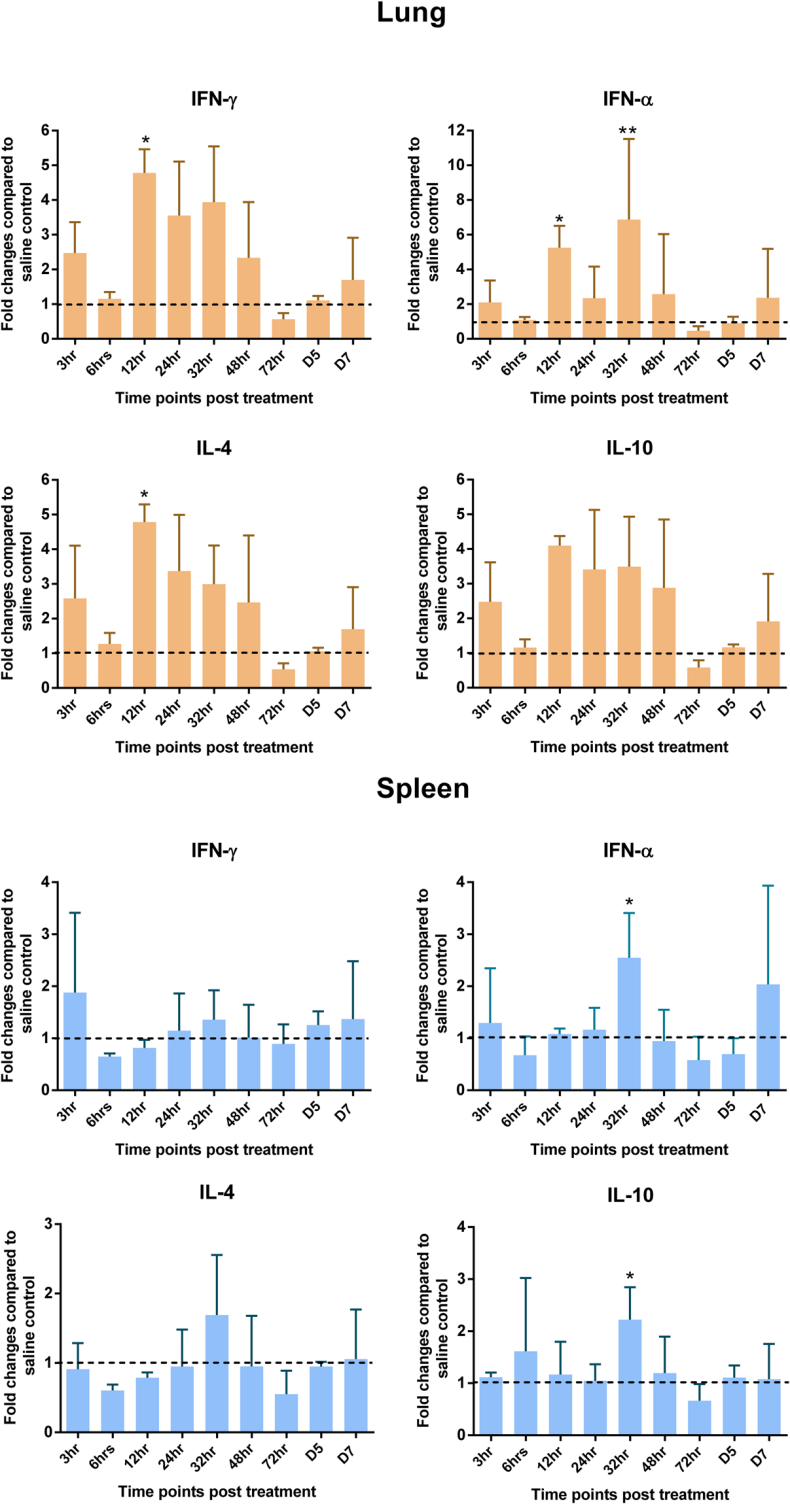
Interferon and regulatory cytokine profile in lung and spleen. MFI of each gene was normalized to the average of HKGs – (Hprt 1 and Tubb 1), then fold changes were calculated against the saline control at each time point and the means were graphed. (Dotted line shows a fold change of one, which indicates no change compared to the control group and the vertical line and horizontal bar show the standard error of mean-SEM). Dunnett’s test following ANOVA testing was used to test for significant differences of gene expression between CpG-ODN group and the saline control group at each time point. Asterisks indicate groups that were significantly different from control group, *P < 0.05 and **P < 0.01.

The mechanism of action of CpG-ODN in these chicks was also elucidated by measuring gene expression patterns of pro-inflammatory (IL-1β, IL-6, LITAF, IL-18), Th1 type (IFN-γ, IFN-α) and Th2 type (IL-4, IL-10) cytokines in spleen and lungs at several hours post CpG-ODN treatment. Fold changes of mRNA levels were 1 or >1 at almost all time points in birds of CpG-ODN treated experimental group compared to the saline control group. Overtime, levels of gene expression were not consistent nor followed a specific pattern. However, most significant differences in gene expression occurred in the 32 hr to 48 hr period after treatment. Normalized gene expression of pro-inflammatory cytokines (IL-18, IL-1β and IL-6 and LITAF) tended to show a relatively higher level in CpG-ODN treated group compared to the saline treated group. The expression of IL-1β and IL-6 in the lungs of chickens was many fold higher compared to spleen at 6 through 48 hr and 12 through 48 hr, respectively. The expression of IL-18 and LITAF mainly increased between 12 through 72 hr and 32 through 72 hr, respectively in both organs (Fig. 2). The gene expression levels of Th1 type cytokines (IFN-γ, IFN-α) and Th2 type cytokines (IL-4, IL-10) were higher in lungs compared to spleens in CpG-ODN treated birds (Fig. 3).

### Flow cytometry analysis

Flow cytometric analysis of spleen and lung cells isolated from CpG-ODN treated birds showed a marked influence of CpG-ODN on antigen presenting cells (APCs) (monocyte/macrophage), expression of costimulatory markers on APCs and T lymphocyte populations.

#### Antigen presenting cells

**Figure 4:**
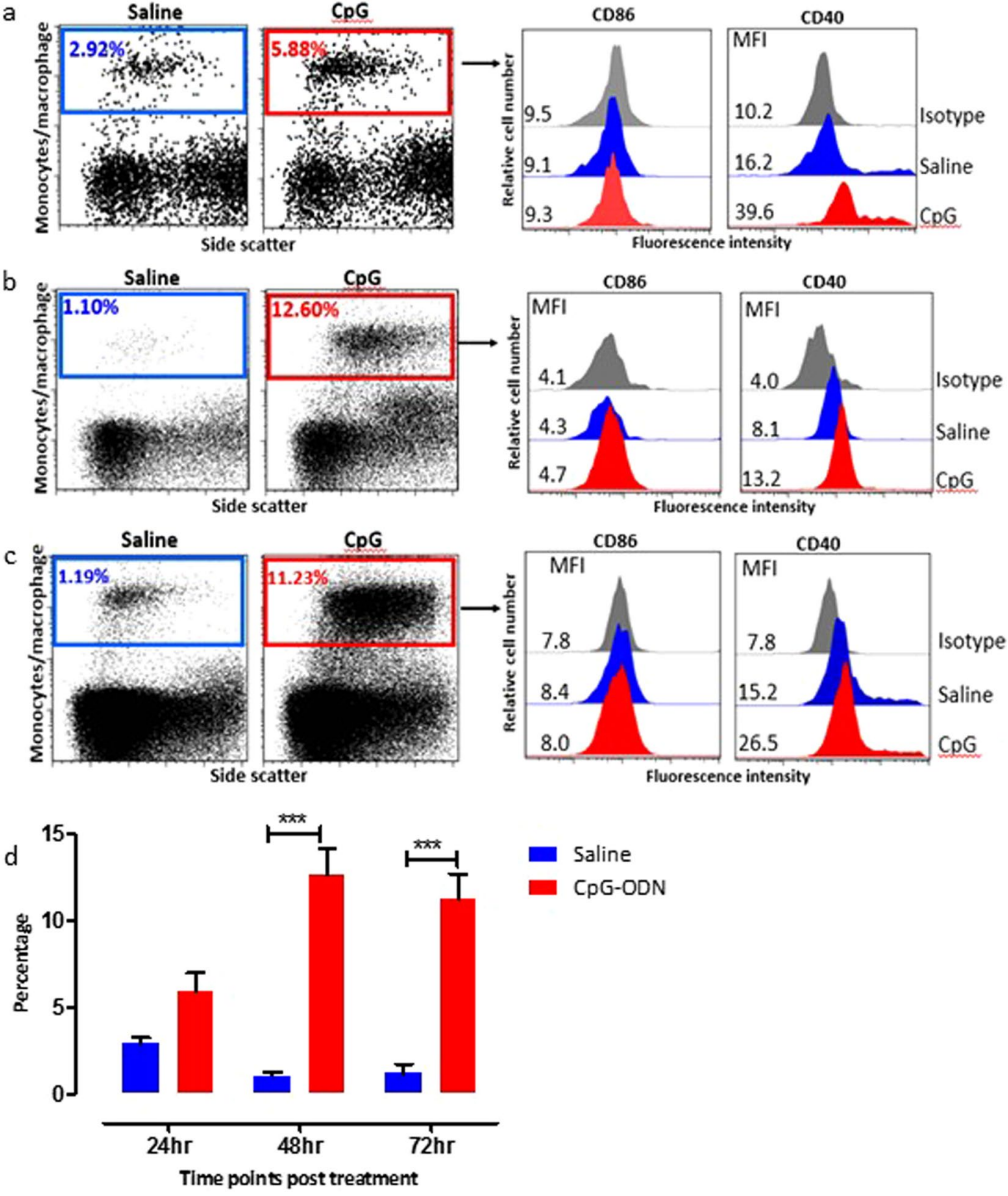
Flow cytometric analysis of spleen cells at 24 hr (**a**), 48 hr (**b**) and 72 hr (**c**) post *in-ovo* injections. (Day 19, 20 and 21 embryo). Macrophages were analyzed (left panels) by gating on monocyte/macrophages population based on forward and side scatter plot. Histogram panels on right indicate the level of CD86 and CD40 (costimulatory molecules found on APCs) expression on the APCs. (**d**) Bar diagram show the mean percentages of monocyte/macrophage APCs following *in-ovo* treatment in spleen. vertical line and horizontal bar show the standard error of mean (SEM), n = 3–4. Two way ANOVA following Bonferroni post-test was done to compare means of APC percentages of CpG-ODN received groups with saline control. Asterisks indicate significant differeces between the groups, ***P < 0.001.

Since the overall cell number was low at 24 hr post treatment, when the chicken embryos were 19 days old, we could only obtain and analyse spleen cells at this time point. CpG-ODN treatment induced a notable increase in the expression of costimulatory molecules (CD40) and the number of APCs (monocyte/macrophage cells) in spleen and lungs compared to saline treated embryos. Compared to number of APCs detected in saline control birds, the percentage of APCs in CpG-ODN treated embryos in spleen at 24, 48 and 72 hrs post treatment were 5.88, 12.6 and 11.2%, respectively (Fig. 4a–d). A considerably higher expression of CD40 costimulatory molecule was observed in APCs in CPG-ODN treated embryos compared to saline treated embryos as indicated by a shift in the MFI to the right (Fig. 4). Even though there was higher expression of CD86 in CpG-ODN group compared to the saline group, no significant difference was observed at any of the time points.

**Figure 5:**
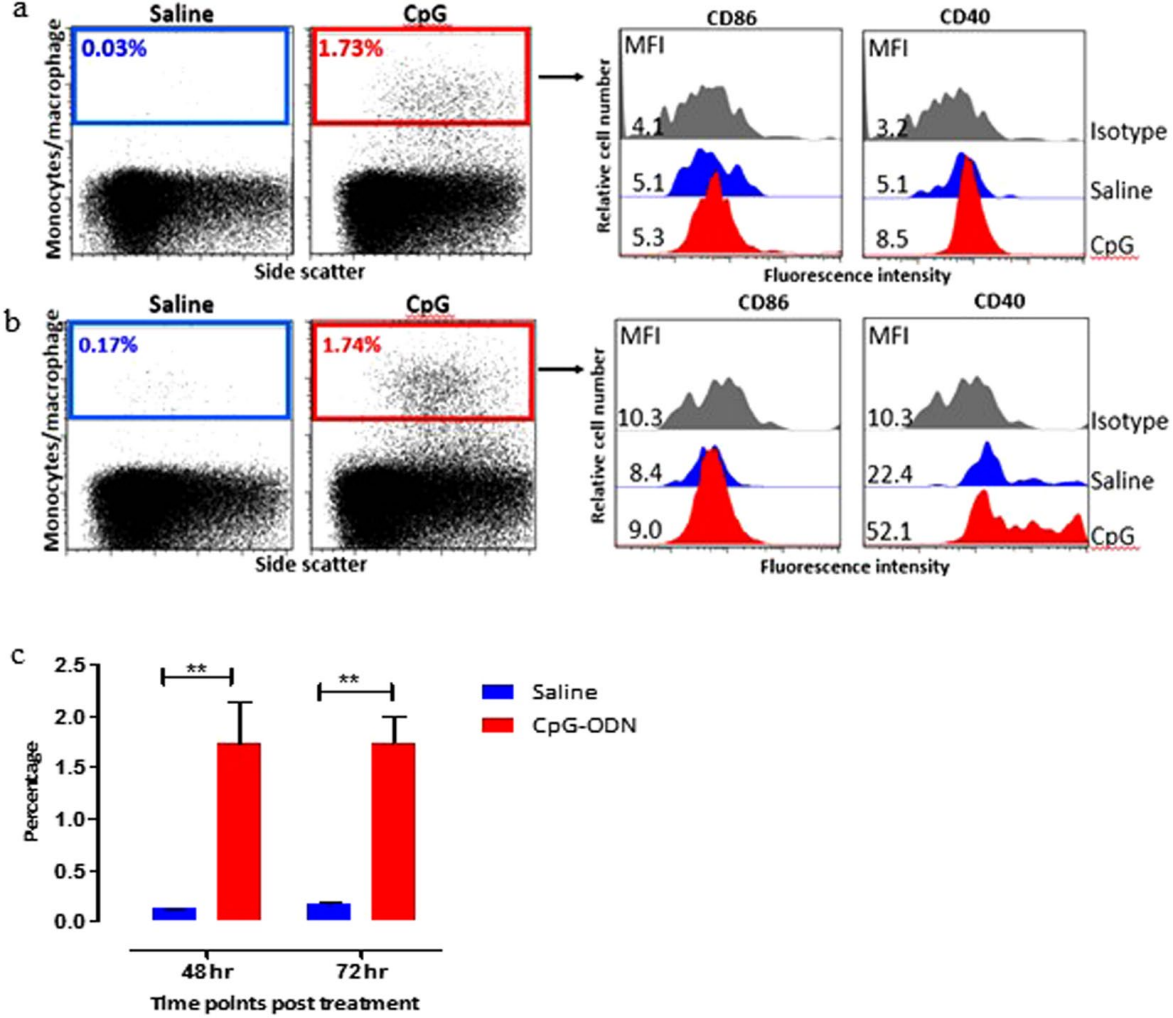
Flow cytometric analysis of lung cells 48 (**a**) and 72 hrs (**b**) post *in-ovo* injections. (Day 20 and 21 embryo). Macrophages were analyzed (left panels) by gating on monocyte/macrophages population based on forward and side scatter plot. Histogram panels on right indicate the level of CD86 and CD40 (costimulatory molecules found on APCs) expression on the APCs. (**c**) Bar diagram show the mean percentages of monocyte/macrophage APCs following *in-ovo* treatment in lung. vertical line and horizontal bar show the standard error of mean SEM, n = 3–4. Two way ANOVA following Bonferroni post-test was done to compare means of APC percentages of CpG-ODN received groups with saline control. Asterisks indicate significant differeces between the groups, **P < 0.01.

A similar trend was observed in the percentage of APCs in the lungs of CpG-ODN treated embryos compared to saline treated embryos at 48 and 72 hrs post *in-ovo* injections. The percentage of APC in the lungs of CpG-ODN treated embryos at 48 and 72 hrs post treatment were 1.73 and 1.74% whereas the percentage of APCs in saline treated birds at 48 and 72 hrs were 0.03 and 0.17% respectively (Fig. 5a and b). CD40 expression was significantly higher in APC in lungs of CpG-ODN treated embryos compared to saline treated embryos and as indicated by a shift in the MFI to the right. Similar to the spleen, no increase in the expression of CD86 costimulatory molecule was observed in APCs in lungs. This striking enrichment of immune cells in the CpG-ODN treated group was noticed at all three time points (24, 48 and 72 hrs post CpG-ODN inoculation) in both spleen and lung.

#### CD8^+^ and CD4^+^ T cells

**Figure 6:**
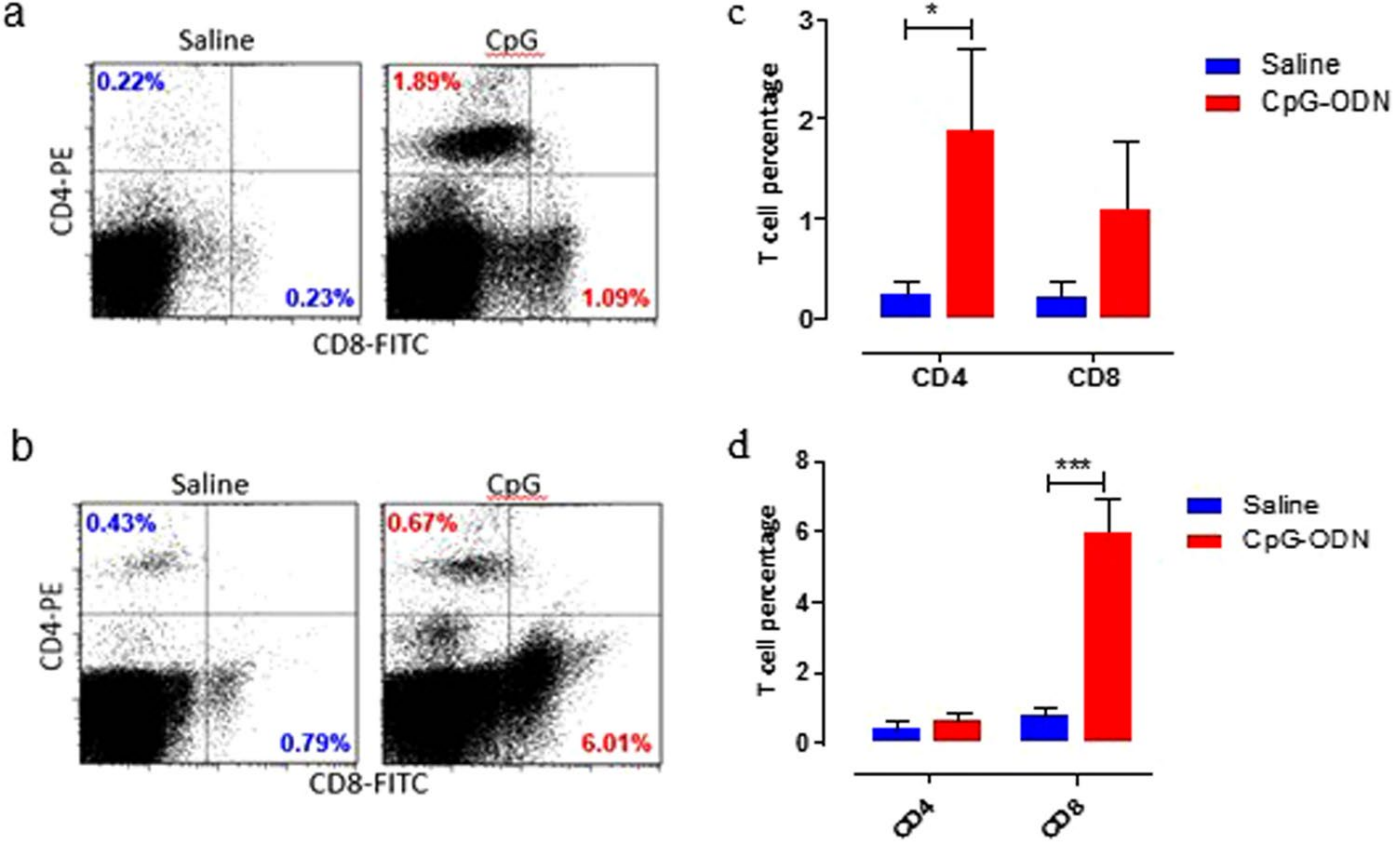
Flow cytometric examination of immune cells in organs. Spleen (**a**) and lung (**b**) T cell populations in day-21 embryo (72 hr post CpG-ODN or saline injection). T lymphocytes were analyzed gating on lymphocytes population based on forward and side scatter plot. CD4^+^ and CD8^+^ T-cells were quantified using PE-labelled mouse anti-chicken CD4 and FITC-labelled mouse anti-chicken CD8 monoclonal antibodies. The bar diagrams (**c**) spleen and (**d**) lung show the mean percentages of CD4^+^ T-cells and CD8^+^ T-cells 72 hr following *in ovo* treatment. Vertical line and horizontal bar show the standard error of mean SEM, n = 3–4. Two way ANOVA following Bonferroni post-test was done to compare means of T cell percentages of CpG-ODN received groups with saline control. Asterisks indicate significant differences between the groups, *P < 0.05, ***P < 0.001.

CD8^+^ and CD4^+^ T cell populations were analysed in spleen and lung at 72 hrs post CpG-ODN injections (21 day old embryos). Both CD8^+^ and CD4^+^ cells were markedly increased in spleen and lung of CpG-ODN treated birds compared to the saline control. The percentage of CD4 and CD8 T cells in spleen of CpG-ODN treated embryos were 1.89% and 1.09%, respectively compared to 0.22% CD4^+^ T cells and 0.23% CD8^+^ T cells in saline control (Fig. 6a). Similarly, the percentage of CD4^+^ and CD8^+^ T cells in lungs of CpG treated embryos were 0.67% and 6.01%, respectively compared to 0.43% CD4^+^ T cells and 0.79% CD8^+^ T cells in saline control (Fig. 6b). Interestingly, while the percentage of CD4^+^ T cells was significantly higher in CpG-ODN group compared to saline in the spleen (Fig. 6c), the percentage of CD8^+^ T cell increase was more prominent in lungs than CD4^+^ T cells (Fig. 6d).

**Figure 7:**
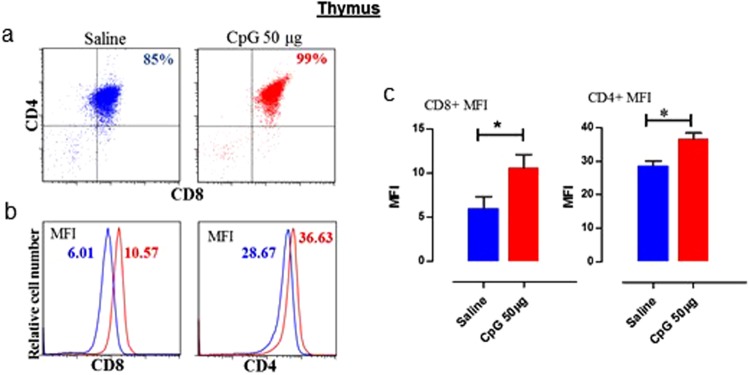
T lymphocytes in the thymus. Flow cytometric analysis of thymus T cell populations of saline control and 50 µg CpG-ODN group (n = 3–4/group) at 72 hr post *in-ovo* injections (day-21 embryo). (**a**) T lymphocytes were analyzed gating on lymphocytes population based on forward and side scatter plot. CD4^+^ and CD8^+^ T-cells were quantified using PE-labeled mouse anti-chicken CD4 and FITC-labelled mouse anti-chicken CD8 monoclonal antibodies. (**b**) Histogram panels indicate the levels of CD4^+^ and CD8^+^ expression on the T cells (the MFI shown in blue indicates saline treated group, and in red indicates the CpG-ODN treated group). (**c**) Bar diagrams show the MFI of CD4^+^ and CD8^+^ expression. Difference of MFI between the two groups were compared using Student-t test with Welch’s correction for unequal variance. n = 3–4, *P < 0.05.

Moreover, to gain insight whether increase in the number of CD4^+^ or CD8^+^ is due to increased differentiation of CD4^+^ or CD8^+^ T cells in thymus, we analysed thymus cells. We observed that the T cell population in thymus was remarkable, where 99% of the total thymus cells were CD4^+^/CD8^+^ double positive at the time of hatch in CpG-ODN treated embryos. In contrast, only 85% the total thymus cells were CD4^+^/CD8^+^ double positive at the time of hatch in saline treated embryos. Both CD4^+^ and CD8^+^ expression levels were significantly higher in CpG-ODN treated group than the saline control group (Fig. 7).

## Discussion

There is strong evidence to suggest that CpG-ODN administration modulates innate and adaptive immune responses in chickens enhancing resistance to several bacterial pathogens including *E*. *coli*^12,24,25^, *Salmonella* Typhimurium^14,28^, *Salmonella* Enteritidis^26,27^, and *Campylobacter jejuni*^37^. The immunoprotective effects of CpG-ODN have also been reported in mice against *Helicobacter pylori*^38^, *Yersinia pestis*^39^, influenza virus^40^, and *Listeria monocytogenes*^41^. These findings suggest that CpG-ODN induces protection in several species against a wide range of pathogens. However, how CpG-ODN treatment confers protection remains poorly understood. The present study was undertaken to gain a better understanding of the mechanisms of CpG-ODN induced host responses that enhance resistance in neonatal chickens to a bacterial pathogen. In this study, we examined how CpG-ODN, delivered by *in ovo* route to18-days old embryonated eggs, protects neonatal chicks, 1-day after the hatch (four days after the CpG-ODN treatment), against a virulent strain of *E*. *coli*. Consistent with our previous studies^12,24,25,42^ in the present study, we found that CpG-ODN treated chicks were significantly protected against *E*. *coli* infection.

During a pathogenic insult, the host immune response is orchestrated by a complex network of cytokines that shape the immune process by regulating cellular communication and function. CpG ODN has been shown to elicit superior cytokine responses in chicken immune cells *in vitro* compared to other TLR ligands (lipopolysaccharide or Pam3CSK4)^43^. Previous studies have suggested that the protection conferred by CpG-ODN in chickens is primarily due to an enhanced expression of cytokines and chemokines^30,35^. Therefore, we designed experiments to evaluate the effects of CpG-ODN administration on the expression profiles of cytokines involved in innate and adaptive immune responses. We examined cytokine expression kinetics of eight cytokine genes (IFN-α, IL-18, IFN-γ, IL-1β, LITAF, IL-4, IL-6, and IL-10) in spleen and lungs of CpG-ODN treated and the saline control. In our study, cytokine expression was measured using QuantiGene Plex 2.0® assay technique, a multiplex assay that can simultaneously measure multiple cytokine genes in a single well of the assay. We observed a substantial upregulation of cytokines following CpG-ODN treatment in spleen and lungs. The mRNA levels of all cytokines tested here tended to be higher in CpG-ODN treated chickens than in the control group at most time points. In agreement with a previous study^44^, we observed higher expression of IFNs following CpG-ODN injections, which was especially notable in the lungs of chickens. IFNs are important cytokines in shaping immune responses against pathogens. IFN-α and IFN-γ have been shown to act synergistically to develop an antiviral state and activate macrophages and nitric oxide secretion^45^. Like Th1 cytokines (i.e., IFNs), Th2 cytokine IL-4 and regulatory cytokine IL-10 were also upregulated following CpG-ODN injections and were also more noticeable in lungs. A previous study reported that CpG-ODN induces a Th1 type of response in chickens^34^. However, in our study, we observed both Th1 and Th2 types of response. This finding is also supported by the recent report that demonstrated both Th1 and Th2 types of cytokine expression in chickens in response to CpG-ODN treatment^30^. Based on present data and previous studies, our data suggest that CpG-ODN in chickens can be used as modulator of both Th1 and Th2 types of cytokine.

Accumulating evidence suggests that pro-inflammatory cytokines play a crucial role in promoting strong immune response against pathogens. Our findings of an enhanced expression of pro-inflammatory cytokines, IL-1β, IL-6, IL-18, and LITAF in lungs and spleen following *in ovo* delivery of CpG-ODN suggests that CpG-ODN promotes inflammatory responses in chickens. Our finding is also supported by recent studies showing upregulation of pro-inflammatory cytokines in chickens following CpG-ODN treatment^37,43^. IL-1β is well known for its induction of IL-6^46^ and in T cell proliferation^47–49^. We found that among all the cytokines investigated in this study, IL-1β was the most upregulated (several fold higher compared to control) cytokine following CpG-ODN administration. Protective effect of pro-inflammatory cytokines is also evident from the study that demonstrated a strong correlation between pro-inflammatory cytokine level and the resolution of *Salmonella* Typhimurium infection in chickens^50^. In the present study, we observed that CpG-ODN treatment led to a more pronounced effect on the cytokine profiles in lungs compared to the spleen, which could suggest that CpG-ODN has a superior effect on the mucosal immune system. Our finding is also supported by recent studies that demonstrated enhanced expression of pro-inflammatory cytokines in response to CpG-ODN in intestinal tissues^30^ and reduced intestinal colonization with *Campylobacter sp*.^37^. These findings suggest that CpG-ODN treatment conditions the chicken immune system against pathogenic invasions at mucosal entry levels. Our data suggest that CpG-ODN enhances mucosal immunity and support the role for CpG-ODN as a potent mucosal adjuvant^30^.

Proinflammatory cytokines such as IL-1β, LITAF, IL-18, and IL-6 are rapidly released upon TLR activation^51^. IL-18 can stimulate T-cell proliferation and IFN-γ release in chickens^52^. IL-18 has also implicated in stimulating TNF-α, IL-1β, IL-8, and IL-6^53^. It is worth mentioning that IL-18 and LITAF remained upregulated in both spleen and lungs of hatched chicks for 72 hr post-CpG-ODN treatment. Importantly, among all the cytokines tested here, LITAF remained upregulated for almost all time points studied and in both spleen and lungs. TNF-α is a multifunctional cytokine that has antitumor, antiviral and antibacterial actions. TNF-α induces upregulation of CD40 and other costimulatory molecules in macrophages and dendritic cells^54^.

In the present study, there were substantial differences in the magnitude and kinetics of cytokine expression between CpG-ODN treated and the saline control. These differences can be ascribed to both TLR signaling and cellular composition as well as the number of cells in the organs investigated. Proinflammatory cytokines such as IL-1β and TNF-α orchestrate the secretion of chemokines and leukocyte cell-surface adhesion molecules, supporting the rapid recruitment of immune cells towards inflammatory area^55–57^. CpG-ODN was shown to enhance cytokine gene expression in various immune cells in chickens, including bursal cells^58^, macrophages^59^, splenocytes^60^, and thrombocytes^61^. Proinflammatory cytokines such as IL-1β, TNF-α, IL-18, and IL-6 are mainly produced by macrophages when stimulated by bacterial products^62^.

Therefore, we next investigated the recruitment kinetics, and activation of macrophages in the immunological niches in spleen and lungs of CpG-ODN treated and saline controls using flow cytometry. Compared to saline control group, flow cytometry data indicated a significantly enhanced enrichment of macrophages in both spleen and lungs of CpG-ODN treated embryo and chicks. It is worth mentioning that the lungs’ immunological niches had very low number of macrophages, sentinel cells that provide the first line of defense against pathogens. These data can explain why chicks are vulnerable to common bacterial infections in their neonatal life^63^, as chicks’ immune systems are still not fully mature at the time of hatch. Thus, our study provides new insights into CpG-ODN-mediated antibacterial mechanisms by demonstrating that *in ovo* delivery of CpG-ODN enriches immunological niches in the immature immune system of the chicken embryo so that pathogenic insults can be tolerated more efficiently at hatch. Maturation of antigen presenting cells (APCs) such as macrophages and dendritic cells involves upregulation of costimulatory molecules such as CD40, CD80 and CD86, and the production of cytokines^64^. Besides, CD40 signaling was shown to activate the APCs and facilitate CD8^+^ T-cell priming^65^ to generate protective CD8^+^ cytotoxic T cell (CTL) immunity^66^. Therefore, we investigated expression of CD86 and CD40 on macrophages in spleen and lungs of CpG-ODN treated or saline controls. Interestingly, we found that macrophages in CpG-ODN treated chicks were expressing significantly high CD40; however, CD86 was not very different than saline control. This interesting observation can be explained by the cytokine profiles in our study. In the present study, we found significantly higher expression of LITAF in CpG-ODN group in spleen and lungs for several days after treatment. The previous study has reported that TNFα induces upregulation of CD40 onto macrophages and dendritic cells^54^. Furthermore, it was reported that TNF-α upregulated CD40 but not CD86 in APCs such as Langerhans cells^67,68^. Thus, prolonged expression of LITAF in our study and upregulation of CD40 but not CD86 on macrophages may implicate LITAF for this observation, which corroborates previous studies on Langerhans cells^67,68^. Thus, we hypothesize that enhanced LITAF in CpG-ODN group could be the reason for increased CD40 in our study. However, further studies are needed to understand better TLR21- LITAF-CD40 axis in chickens.

We also examined the T-cell compartment in spleen and lungs of CpG-ODN treated and saline controls chicks that hatched 72 hr post-CpG-ODN treatment. Flow cytometry revealed significantly high number of both CD4^+^ T and CD8^+^ T cells in lungs and spleen of CpG-ODN treated group. These data suggest that CpG-ODN promotes the enrichment of T-cell immunological niches both in lymphoid (spleen) and non-lymphoid (lungs) organs in chickens. The increase in the number of immune cells in spleen and lungs can be due to either enhanced recruitment of T cells or increase thymic out (generation of T cells in the thymus) or both. Given that in thymus T cells are generated as CD4(+) and CD8(+) double positive cells and after thymic selection T cells egress thymus as either CD4^+^ T cells or CD8^+^ T cells. Therefore, we harvested thymus from CpG-ODN treated and saline control chicks to investigate the expression of CD4 and CD8 in thymocytes as an indicator of thymopoiesis. Interestingly, we found significantly high expression of both CD4 and CD8 in the thymocytes of CpG-ODN treated group. A previous study in mice demonstrated that CPG-ODN treatment resulted in a systemic antigen-independent expansion of both CD4^+^ and CD8^+^ T cell subsets and suggested that increase in T cell number was the consequence of reduced T cell death in CpG-ODN treated mice^69^. However, further studies are needed to investigate the role for a reduced T cell death or potentially increased thymic output in neonatal chicks following CpG-ODN treatment to explain the increased T cell numbers in our study. Previous studies using transgenic mice reported that TNF-α is constitutively expressed in the thymus^70^ and promotes murine^71^ and human^72^ T cell development. In our study, LITAF expression pattern stands out in the cytokine profiles of spleen and lungs. Therefore, we hypothesize that CpG-ODN induced LITAF may have more significant roles in enriching immune compartments such as spleen and lungs with various sentinel immune cells; consequently, neonatal chicks become better equipped to fight against pathogenic insults.

In conclusion, the present study has demonstrated for the first time that *in ovo* delivery of CpG-ODN provides protection against *E*. *coli* infection in chickens by enriching immunological niches in spleen and lungs that enhances immunocompetence of neonatal chicks to tackle pathogenic insults. Furthermore, our data highlight the importance of LITAF in CpG-ODN induced immunoprotective mechanisms and suggest that future studies on TLR21-LITAF-mediated immune enrichment axis will help in harnessing the full therapeutic potential of CpG-ODNs against diseases.

## Materials and Methods

### Synthetic CpG-ODN

The sequence of CpG-ODN (class B CpG 2007) 5′-TCGTCGTTGTCGTTTTGTCGTT-3′ used in the study was produced with a phosphorothioate backbone (Operon Biotechnologies, Inc. Huntsville, AL).

### Bacteria

The bacterial strain for challenge was obtained from a field isolate of *E*. *coli* from a turkey with septicemia as described in previous studies^12,24^. This *E*. *coli* was serogroup O_2_, non-hemolytic, serum-resistant, produced aerobactin, with a K1 capsule and Type 1 pili. Aliquots of bacteria were stored at −80 °C in 50% brain–heart infusion broth (BHI; Difco, Detroit, MI) supplemented with 25% (w/v) glycerol (VWR Scientific, Inc., Montreal, Quebec). Bacteria used for challenge were cultured on columbia sheep blood agar plates for 18–24 hr at 37 °C. One colony was added to 100 ml of Luria broth in a 250 ml Erlenmeyer flask. The culture was grown at 37 °C for 16–18 hrs with shaking at 150 rpm. Stationary phase culture contained approximately 1 × 10^9^ colony forming units (cfu) of bacteria per ml. The cultures were further diluted in sterile saline so the concentration of bacteria required for challenge (1 × 10^5^ or 1 × 10^4^ cfu/bird) was obtained. Viable bacterial counts were determined by plating serial dilutions of the diluted culture in duplicate on Columbia sheep blood agar plates, incubating for 18–24 hrs at 37 °C; then counting the colonies.

### Chicken embryos

For all experiments (*E*. *coli* challenge study, gene expression and flow cytometry analysis), fertilized hatching eggs were obtained from a commercial broiler breeder operation in Saskatchewan, Canada. Eggs were incubated until hatch at the Animal Care Unit (ACU) at the Western College of Veterinary Medicine, University of Saskatchewan. This work was approved by the University of Saskatchewan’s Animal Research Ethics Board and adhered to the Canadian Council on Animal Care guidelines for humane animal use.

### Animal model for *E*. *coli* challenge

Hatched chicks were allocated into an animal isolation room at the ACU. An identification tag was placed on their neck in order to identify birds that received in-ovo CpG-ODN or saline. Chicks of both groups were housed in the same condition and were kept on floor with wood shavings as litter. Water and commercial broiler ration were provided *ad libitum* throughout the trial. The animal room was ventilated with filtered, non-recirculated air at a rate of 10–12 changes/hr. In addition, air pressure differentials and strict sanitation were maintained in this isolation facility. One day post-hatch, the birds received either 1 × 10^4^ or 1 × 10^5^ cfu’s of stationary - phase *E*. *coli*, in a total volume of 250 μl per bird, by subcutaneous injection in the neck, resulting in *E*. *coli* septicemia. In this model, *E*. *coli* septicemia with airsacculitis, pericarditis, perihepatitis or polyserositis develops in 60–90% of birds that are not protected by treatment intervention. The two different doses of *E*. *coli* were given to groups of birds to simulate field conditions where all birds are not exposed to a consistent dose of *E*. *coli* in a barn. Birds were assessed three times daily at the critical stage (first four days post challenge) and twice thereafter up to 8 days post challenge. Birds were observed for clinical signs and each individual bird was assigned a daily clinical score: 0 = normal; 0.5 = slightly abnormal appearance, slow to move; 1 = depressed, reluctant to move; 1.5 = reluctant to move, may take a drink and peck some; 2 = unable to stand or reach food or water; and 3 = found dead. Birds that received a clinical score of 2 were humanely euthanatized by cervical dislocation. Chicks that were found dead or euthanatized were necropsied immediately. On day 8 post *E*. *coli* challenge, the remaining birds were euthanatized by cervical dislocation. Bacterial swabs were taken immediately after necropsy from the air sacs and cultured on Columbia sheep blood agar using a typical method of inoculation and streaking on four quadrants of the plate of medium. A semi-quantitative estimate of *E*. *coli* isolation was conducted on Columbia sheep blood agar by the quadrant streaking method in which bacterial colonies thinning occurs as streaking goes clockwise from quadrant 1 to quadrant 4. Growth on these plates were recorded on a scale from 0 to 4+, where 0 = no growth; 1+ = growth of bacteria on the quadrant 1 only; 2+ = growth of the bacteria on quadrants 1 and 2; 3+ = growth of bacteria on quadrants 1, 2, and 3; and 4+ = growth of bacteria on all quadrants 1–4.

### Cells for flow cytometry

Cell preparation and antibody staining for flow cytometry was done as previously described with some modifications^73–75^. Briefly, spleen, lung and thymus tissues were collected at 24, 48 and 72 hr post *in ovo* injection from chicken embryos and processed for cell isolation (thymus only at 72 hr). Each spleen was gently pushed through a metal strainer by manual pressure to obtain a single cell suspension with ~3 ml of phosphate buffered saline (PBS) and collected in a 15 ml centrifuge tube. For lung and thymus, each tissue was manually dissected and incubated with ~1 ml of collagenase type 4 (Sigma-Aldrich, St. Louis, Missouri, USA) (1 mg/ml) dissolved in Dulbecco’s Modified Eagle Medium for 30 minutes in 37 °C. After incubation these tissues were pushed through a metal strainer to obtain a single cell suspension and washed twice with PBS. Then all spleen, lung and thymus cells were incubated with red blood cell lysis buffer to lyse red blood cells. Following three washes with wash buffer (PBS containing 2% fetal bovine serum and 0.1% sodium azide), the cells were used for the flow cytometry after staining with appropriate antibodies. In flow cytometry, lymphocyte gate for T cells was determined by staining cells with anti-CD3 antibody and then back gating on forward and side scatter plot. Similarly strategy was applied to identify macrophages gate on forward and side scatter plot.

### Antibodies for flow cytometry

Monoclonal antibodies against chicken monocyte/macrophages (mouse anti-chicken monocyte/macrophages-PE), CD4 (mouse anti-chicken CD4-PE) and CD8 (mouse anti-chicken CD8α-FITC) were purchased from Southern Biotechnology (Birmingham, Ala, USA). Mouse anti-chicken CD40 and mouse anti-chicken CD86 monoclonal antibodies were used as primary antibodies (purchased from Bio-Rad, Raleigh, NC, USA). Anti-mouse-FITC IgG antibody was used as secondary antibody. Goat anti-mouse IgG, Streptavidin-PerCP/Cy5.5 and Mouse IgG1 isotype control were purchased from Bio Legend (San Diego, CA, USA).

### Experimental design

#### Delivery of CpG-ODN by in ovo route

Eighteen days old embryonated eggs were injected with 50 µg of synthetic CpG-ODN dissolved in sterile pyrogen-free saline, in a total volume of 100 µl/egg (n ≈ 90 for all three experiments) or 100 µl of sterile saline (~n = 90). The air cell side of each egg was cleaned with an alcohol wipe and injections were administered into the amniotic cavity through the air cell using a 22-gauge, 1-inch hypodermic needle. Following injections, a drop of melted wax was placed on the pore created to seal the egg. All eggs were then transferred to the incubator until hatch or taken for tissue sample collection.

#### *E. coli* challenge

Four days after *in ovo* injections (day-1 post-hatch), either 1 × 10^4^ or 1 × 10^5^ cfu’s of a virulent strain of *E*. *coli* was inoculated subcutaneously in the neck in all remaining (after sample collection for flow cytometry and gene analysis) hatched birds [bird numbers per group; CpG-ODN 50 µg: n = 32; saline: n = 36]. Clinical signs, pathology, bacterial isolations from the air sacs, and mortality were observed for 8 days following challenge with *E*. *coli*.

#### Sample collection

For QuantiGene Plex assay- spleen and lung samples from three embryos or newly hatched chicks per group were collected at 10 time points (0, 3, 6, 12, 24, 32, 48, 72 hrs, day 5 and day 7) post CpG-ODN inoculation in 1.5 ml tubes and flash frozen in dry ice until stored in −80 °C. For flow cytometry*-*three embryos or newly hatched chicks from each group were humanely euthanized at 24, 48 and 72 hr post *in ovo* injections by cervical dislocation and necropsied for spleen, lung and thymus tissue collection (thymus only at 72 hr post *in ovo* injections).

#### Gene expression analysis

The mRNA gene expression of IFN-α, IL-18, IFN-γ, IL-1β, LITAF, IL-4, IL-6 and IL-10 cytokine genes in the spleen and lung were measured by QuantiGene Plex 2.0® technique using commercially available probes for Avian cytokines (Panomics/Affymetrix Inc., Fremont, CA, USA). The genes of interest and their accession numbers are listed in Table 1.

**Table 1:** Genes of interest.

Gene	Gene name	Accession Number
IL 18	Interleukin 18	NM_204608
IL 4	Interleukin 4	NM_001007079
IFNα	Interferon alpha	NM_205427
IL 10	Interleukin 10	NM_001004414
IFNγ	Interferon gamma	NM_205149
IL 1β	Interleukin 1, beta	NM_204524
Hprt 1*	Hypoxanthine-guanine phosphoribosyl transferase 1	NM_204848
LITAF	Lipopolysaccharide-induced TNF factor	NM_204267
Tubb 1*	Tubulin, beta 1	NM_205445
IL 6	Interleukin 6	NM_204628

(*Housekeeping genes- HKGs).

Frozen spleen and lung tissues were processed and tissue homogenates were prepared following the manufacturer’s instructions with some modifications. Briefly, 5 mg of tissue was excised, manually broken down and added to 300 µl of homogenization solution and 3 µl of Proteinase K. The tissue lysate was digested at 65 °C to release the mRNA and centrifuged to precipitate the debris. The supernatant of tissue lysates were collected and stored in −80 °C if not used the same day. The oligonucleotide capture probes for mRNA and label probes were designed by the manufacturer as requested. The tissue homogenates were added to a 96-well plate (40 µl/well) that was pre-loaded with 20 mL of the capture reagent and the respective probe set. After overnight hybridization at 54 ± 1 °C, hybridizations with bDNA pre-amplifier 2.0, bDNA amplifier 2.0, biotinylated label probe and finally substrate were subsequently carried out according to the manufacturer’s instructions. Luminescence was quantified using a Luminex instrument (Bio-Rad, USA). Signals, which is the mean fluorescence intensity (MFI) generated from each bead, are proportional to the amount of each mRNA captured on the surface of each generated specific probe set^76^. The expressions of these genes were normalized with the expression of hypoxanthine-guanine phosphoribosyl transferase 1 (Hprt 1) and tubulin beta 1 (Tubb1) genes which were used as housekeeping genes (HKGs). For gene expression analysis, first, the MFI data of each gene was normalized by dividing with the average of Hprt 1 and Tubb 1HKGs. Then, using normalized MFI data fold changes were calculated in CpG-ODN group by determining how many folds expression compared to saline control group at each time point.

#### Flow cytometry

The lung and spleen cell populations were stained for the presence of monocyte/macrophages, CD4^+^ and CD8^+^ T cells. The monocyte/macrophages were further analysed for the expression of maturation markers (CD40 and CD86). Briefly, ~5 × 10^5^ cells were incubated with mouse anti-chicken monocyte/macrophage phycoerythrin (PE) antibody at 4 °C for 30 min for detecting APCs. For determining the maturation state of monocyte/macrophages, the cells from previous step were washed three times and incubated with either mouse anti-chicken CD40 or CD86 primary antibodies separately at 4 °C for 30 min. After three washings with PBS, the cells were stained with PerCP/Cy5.5 goat anti-mouse IgG secondary antibody at 4 °C for 30 min. Another set of ~5 × 10^5^ cells were also incubated with mouse anti-chicken CD8 (FITC) and CD4 (PE) together at 4 °C for 30 min to determine CD4^+^ and CD8^+^ T cells. Finally, the washed cells were suspended in 300 µl buffer in flow tubes and processed for flow cytometric analysis. Flow cytometry data were acquired by Epics XL (Beckman Coulter) and FACS Caliber (BD Bioscience), and data were analyzed with FlowJo software (Tree Star).

### Statistical analysis

Survival pattern, cumulative clinical scores (CCS), bacterial percentages and cell populations from flow cytometry analysis were graphed and analyzed with the use of Prism (Prism 5.0, GraphPad Software Inc., San Diego, CA) with a significance level of P < 0.05. The survival patterns were compared using the log-rank test and chi-square statistic. For calculating CCS, the clinical score for each bird was summed over the 8-day observation period and the significance of differences among groups were tested with Kruskal-Wallis nonparametric analysis of variance. Dunnett’s test following ANOVA testing was used to test for significant differences of gene expression between the CpG-ODN group and the saline control group at each time point. For testing difference of APC percentages, CD4^+^ and CD8^+^ expression between groups, we used a two way ANOVA followed with Bonferroni post-test and Student-t test with Welch’s correction for unequal variance, with a significant difference of P < 0.05.
